# Veal Liver as Food Vehicle for Human *Campylobacter* Infections

**DOI:** 10.3201/eid2406.171900

**Published:** 2018-06

**Authors:** Colette Gaulin, Danielle Ramsay, Réjean Dion, Marc Simard, Céline Gariépy, Éric Levac, Karon Hammond-Collins, Maude Michaud-Dumont, Mélanie Gignac, Marc Fiset

**Affiliations:** Ministère de la Santé et des Services Sociaux, Québec City, Québec, Canada (C. Gaulin, M. Fiset);; Ministère de l’Agriculture des Pêcheries et de l’Alimentation du Québec, Québec City (D. Ramsay, M. Michaud-Dumont, M. Gignac);; Laboratoire de Santé Publique du Québec, Sainte-Anne-de-Bellevue, Québec City (R. Dion);; Institut National de Santé Publique du Québec, Québec City (M. Simard);; Centre Intégré de Santé et de Services Sociaux de la Montérégie-Centre, Longueuil, Québec (C. Gariépy, É. Levac);; Agence de la Santé Publique du Canada, Longueuil (K. Hammond-Collins)

**Keywords:** epidemiologic studies, *Campylobacter* infections, campylobacteriosis, foodborne illnesses, risk factors, veal liver, enteric infections, food safety, bacteria, Canada

## Abstract

A matched case–control study in Quebec, Canada, evaluated consumption of veal liver as a risk factor for campylobacteriosis. *Campylobacter* was identified in 28 of 97 veal livers collected concurrently from slaughterhouses and retailers. Veal liver was associated with human *Campylobacter* infection, particularly when consumed undercooked.

Recent investigations conducted in Quebec, Canada, after an increased number of sporadic campylobacteriosis illnesses suggested that consumption of veal liver may be a risk factor for campylobacteriosis. Many of the persons infected reported eating veal liver, and many of those had eaten it pink or undercooked. The association between campylobacteriosis and the consumption of meat products, including chicken liver and offal from different animal species, has been previously described (*1*–*5*). We designed an epidemiologic study to examine the relationship between veal liver consumption and campylobacteriosis.

## The Study

We conducted a matched case–control study to examine a potential association between veal liver consumption and campylobacteriosis, using salmonellosis cases as controls. The study began in September 2016 and continued for 9 months. Salmonellosis and campylobacteriosis cases are reportable in Quebec; we selected all subjects from the provincial reportable disease registry. We used a systematic sampling method to select every fifth reported campylobacteriosis case-patient >45 years of age. We paired each campylobacteriosis case-patient with 1 salmonellosis case-patient by age group (45–64 and >65 y) and sex; both infections were confirmed by fecal culture. We matched case-patients if the salmonellosis sample was collected within a window of 7 days before to 60 days after the campylobacteriosis sample was collected. Inclusion criteria for cases and controls were infection that was sporadic and domestically acquired. Exclusion criteria were co-infection with another pathogen, being part of a recognized outbreak, or contact with a gastroenteritis case-patient <10 days before illness.

We administered a structured questionnaire by telephone to collect information on exposures in the 7 days before illness onset. Exposures were consumption of meat and unpasteurized milk products, contact with animals, drinking and recreational water exposures, and occupational exposures. In particular, we investigated consumption of a variety of livers and the degree to which they were cooked. We conducted matched univariate and multivariate analysis to estimate odds ratios (OR) for each exposure.

In addition, we collected samples of veal, chicken, pork, and beef livers from slaughterhouses and retail stores in Quebec between October 2014 and March 2017. We tested each liver specimen for the presence of *Campylobacter*, *Salmonella*, and *Escherichia coli* O157:H7 by using standardized methods (*6*,*7*).

We matched a total of 112 campylobacteriosis cases to salmonellosis cases. We found no significant statistical difference in the age or sex distribution of retained cases or controls and the excluded patients. The species of *Campylobacter* were *C. jejuni* (79.5%), *C*. *jejuni/coli* undifferentiated (3.6%), *C. coli* (0.9%), other (1.8%), and not identified (14.3%). Among campylobacteriosis case-patients, 42 (37.5%) consumed veal liver and 29 (69.0%) ate it undercooked.

Only the consumption of veal liver and having contact with farm animals were statistically significantly associated with campylobacteriosis (Table 1). After applying the Bonferroni correction to adjust for multiple comparisons (0.05 level of significance divided by 45 variables tested yields α = 0.001), only veal liver consumption remained as a statistically significant exposure (matched OR 9.50, 95% CI 3.39–26.62; p = 0.000001).

**Table 1:** Results of univariate matched analysis for patients with campylobacteriosis or salmonellosis, Quebec, Canada, September 2016–May 2017*

Exposures	No. pairs						Matched odds ratio†		p value§
e	f	g	h	Total	Estimate	95% CI‡
Liver									
Chicken liver	0	4	1	104	109		4.00	0.45–35.79	0.2
Pork liver	0	2	5	102	109		0.40	0.08–2.06	0.3
Beef liver	0	3	3	105	111		1.00	0.20–4.96	1.0
Veal liver	4	38	4	62	108		9.50	3.39–26.62	0.000001
Lamb liver	0	3	0	109	112		Undefined	Undefined	1.0
Poultry									
Breaded chicken	10	15	18	64	107		0.83	0.42–1.65	0.6
Ground chicken	0	3	10	95	108		0.30	0.08–1.09	0.07
Whole chicken	44	35	22	8	109		1.59	0.93–2.71	0.09
Pork									
Ham	27	26	26	29	108		1.00	0.58–1.72	1.0
Bacon	8	23	27	50	108		0.85	0.49–1.49	0.6
Ground pork	4	12	14	76	106		0.86	0.40–1.85	0.7
Beef									
Ground beef	47	25	24	9	105		1.04	0.60–1.82	0.9
Roast beef	3	22	15	66	106		1.47	0.76–2.83	0.3
Beef steak	20	21	25	39	105		0.84	0.47–1.50	0.6
Veal									
Ground veal	0	8	13	86	107		0.62	0.26–1.49	0.3
Veal escalope	0	8	2	98	108		4.00	0.85–18.84	0.08
Unpasteurized milk products									
Raw milk	0	2	1	107	110		2.00	0.18–22.06	0.6
Raw-milk cheese	0	10	4	93	107		2.50	0.78–7.97	0.1
Water exposures									
Drinking water from source other than aqueduct	3	12	12	81	108		1.00	0.45–2.23	1.0
Animal exposures									
Dog or cat	24	23	22	38	107		1.05	0.58–1.88	0.9
Farm animal	0	10	1	100	111		10.00	1.28–78.12	0.03
Work in contact with animals	0	8	2	102	112		4.00	0.80–38.67	0.07

*Results by matched analysis: e, campylobacteriosis case exposed, salmonellosis case exposed; f, campylobacteriosis case exposed, salmonellosis case not exposed; g, campylobacteriosis case not exposed, salmonellosis case exposed; h, campylobacteriosis case not exposed, salmonellosis case not exposed.  †By McNemar method. ‡Lower and upper limits determined by McNemar or exact method. §By Wald or Fisher exact test, bilateral.

Among veal liver consumers, adequate cooking (e.g., well-cooked vs. pink or rare, on the basis of the participant’s subjective observation) was protective. Specifically, 13 (30.2%) of 43 case-patients versus 6 (85.7%) of 7 controls ate their veal liver well-cooked (unmatched OR 0.07, 95% CI 0.002–0.72; p = 0.02). Multivariate analysis using logistic regression confirmed that a statistically significant association between the consumption of veal liver and campylobacteriosis remained when all other exposures were included as covariates. Although we conducted this study among persons ≥45 years of age, it is reasonable to assume that eating veal liver, especially undercooked, would also carry risk for younger persons.

We sampled 339 veal, pork, chicken, and beef livers collected from 138 retailers and 16 slaughterhouses. When we evaluated all livers collected at these locations, we detected *Campylobacter* in 28.0% of veal livers, 22.2% of pork livers, 36.8% of chicken livers, and 19.1% of beef livers (Table 2). We detected *Salmonella* more frequently in chicken livers (22.1%) and pork livers (19.1%) than in veal livers (3.1%); we did not detect *Salmonella* in beef livers. We rarely identified *E*. *coli* O157:H7 in livers of any kind. The proportion of contaminated livers differed between animal species and also with respect to location of sampling. A higher proportion of veal livers (35.7%) collected from retailers were contaminated by *Campylobacter*, compared with veal livers collected from slaughterhouses (16.2%). We observed the reverse for chicken and pork livers. The reason for these variations is unclear at this time, but this finding may be an artifact resulting from the relatively small number of samples taken at each location.

**Table 2:** Animal livers collected from retailers and slaughterhouses and percentage positive for *Campylobacter*, *Salmonella*, and *Escherichia coli* O157:H7, Quebec, Canada, October 2014–March 2017

Source and pathogen	Veal livers			Pork livers			Chicken livers			Beef livers		Total
	No.	% Positive		No.	% Positive		No.	% Positive		No.	% Positive	
Retailer	59			27			19			41		146
* Campylobacter*		35.7			16.7			10.5			28.2	
* Salmonella*		5.1			44.4			32.0			0	
* E. coli* O157:H7		0			0			0			0	
Slaughterhouse	38			41			58			56		193
* Campylobacter*		16.2			25.6			45.6			12.7	
* Salmonella*		0			2.4			19.0			0	
* E. coli* O157:H7		0			2.4			0			0	
Total	97			68			77			97		339
* Campylobacter*		28.0			22.2			36.8			19.1	
* Salmonella*		3.1			19.1			22.1			0	
* E. coli* O157:H7		0			1.5			0			0	

Cattle are a well-known reservoir for a variety of *Campylobacter* species, such as *C. jejuni*, *C. coli*, and *C. fetus* (*8*,*9*). *Campylobacter* species have been isolated from beef intestinal contents and also from beef bile, bile ducts, gallbladder, and liver (*10*–*14*). The gallbladder and bile contain substances that have a chemoattractant effect on *C. jejuni*, which explains the presence of *Campylobacter* within the biliary tract (*10*,*15*). Liver contamination varies between animal species (*10*–*14*). Chicken liver, for example, can be contaminated by *Campylobacter* and S*almonella* and has been the source of several outbreaks (*3*,*4*,*11*,*13*). Because few case-patients consumed livers from other animal species during our study, we were not able to identify any substantial risks associated with those exposures.

Because livers may be collected from several animals and stored together, they may be contaminated during the evisceration process or by cross-contamination (*11*). Both the external and internal tissue of a liver may be contaminated with *Campylobacter*, and inadequate cooking may not fully inactivate *Campylobacter* and *Salmonella* (*10*,*11*), which is a cause for concern because ≈70% of the patients with campylobacteriosis who consumed veal liver in our study reported eating it undercooked. We did not examine possible cross-contamination of foods and surfaces and the host-related factors that may increase the risk for enteric diseases.

## Conclusions

Our study identified a strong and statistically significant association between the consumption of veal liver and sporadic, domestically acquired campylobacteriosis among persons >45 years of age in Quebec. We found that adequate cooking of veal liver mitigates the risk of infection. We detected *Campylobacter* in almost one third of veal livers we sampled from slaughterhouses and retail stores, which supports our finding that veal liver consumption is a risk factor for campylobacteriosis. In light of these results, we recommend the dissemination of safe food handling practices for veal liver and other offal for retailers, food establishments, slaughterhouses, and the general public.
